# Radiation damage as a source of information

**DOI:** 10.1107/S2052520624000908

**Published:** 2024-01-29

**Authors:** Elena V. Boldyreva

**Affiliations:** a Novosibirsk State University, Pirogova Street 2, Novosibirsk, 630090, Russian Federation; b Boreskov Institute of Catalysis SB RAS, Lavrentiev Avenue 5, Novosibirsk, 630090, Russian Federation

**Keywords:** radiation damage, thermal expansion, molecular crystals, radiation-induced strain

## Abstract

The structural strain induced by temperature (‘phonon pressure’) and radiation damage (‘defect pressure’) is not necessarily correlated because of different underlying structural mechanisms. Here synchrotron experiments may provide new and yet unexplored opportunities. A recent publication by McMonagle *et al*. [(2024), *Acta Cryst*. B**80**, 13–18] is an excellent illustration of this.

It has long been known that X-ray radiation can damage samples. This phenomenon was extensively investigated in protein crystallography as something unwanted and detrimental for data quality. The main efforts were focused on developing experimental strategies that minimize the damage (Garman, 2010 ▸; Bourenkov & Popov, 2010 ▸). That radiation damage has been observed much more in macromolecular crystallography than for small-molecule samples can be attributed not only to the higher sensitivity of macromolecular crystals to radiation but also to the fact that synchrotron radiation was used much more when studying the crystals of biomolecules than when studying small-molecule crystals.

The advent of new synchrotron sources with increased brightness made synchrotron experimentation very attractive for structural research. As more synchrotron experiments are now performed with small-molecule crystals, radiation damage is becoming more and more frequently reported for these systems too (Christensen *et al.*, 2019 ▸; Bogdanov *et al.*, 2021 ▸; Collings, 2021 ▸; Collings & Hanfland, 2022 ▸). Radiation damage may affect structural data and lead to wrong conclusions on the properties of materials. Serious damage is disturbing, but at least it can be easily detected both by watching for a single crystal with a hole burnt in it by the beam (Fig. 1 ▸) and/or by poor quality of diffraction data.

Radiation damage may be less obvious and easily overlooked, especially during the early stages of the experiment, when the data as well as refined structural parameters and *R* factors look ‘normal’ at first sight. Data collection at a single temperature may reveal no suspicious effects. Multi-temperature data collections starting with the highest temperature and then decreasing may suggest that something ‘strange’ happens, if structural data deteriorate and atomic displacement parameters (ADPs) increase on cooling. If a negative bulk thermal expansion is observed, this also may look suspicious. Such is the case with glycinium phosphite (Bogdanov *et al.*, 2021 ▸). Should the same series of multi-temperature data be collected on heating the crystal from the lowest temperature upwards, no radiation damage effect would be, most likely, even suspected, since an increase in the ADPs, a slight deterioration of structural data and positive thermal expansion would look natural. At the same time, one may note that in some rare cases even a structural contraction on cooling may be masked by structural contraction due to radiation damage (Coates *et al.*, 2021 ▸; Boström *et al.*, 2022 ▸).

What really helped to detect the radiation damage in glycinium phosphite was a comparison of the synchrotron data with the results of the low-dose measurements at a laboratory source. The differences in the temperature dependencies of the unit-cell parameters and volume obtained in a synchrotron experiment and at a laboratory source were really striking and convincing (Fig. 2 ▸). The final proof of the radiation-induced structural expansion was obtained later in additional isothermal experiments (Fig. 3 ▸) (Bogdanov *et al.*, 2022 ▸).

The earlier work by Bogdanov *et al.* (2021 ▸) has not only illustrated how important it may be that a synchrotron experiment is compared with that at a laboratory source. Bogdanov *et al.* have suggested that the radiation damage may be considered as a tool to tune structural disorder and associated properties, and here synchrotron experiments with the additional option of data collections at different energies may provide new and yet unexplored opportunities.

A recent publication by McMonagle *et al.* (2024 ▸) provides an excellent illustration of this. The authors have compared the radiation-induced strain in a series of compounds with thermal expansion of the same crystals. A second-rank tensor of radiation-induced lattice strain was proposed to characterize the structural susceptibility of the crystal to radiation. Such tensors were calculated and compared with the corresponding tensors of thermal expansion (Fig. 4 ▸).

The associated eigenvalues and eigenvectors showed that for Hg(NO_3_)_2_(PPh_3_)_2_ and Hg(CN)_2_(PPh_3_)_2_ [PPh_3_ = tri­phenyl­phosphine, P(C_6_H_5_)_3_; Ph = phenyl, C_6_H_5_] the two tensors were not the same and therefore probed truly different structural responses, whereas for BiPh_3_ they were quite similar.

The authors emphasize that at the level of elastic response, radiative expansivity relates to deformations induced by the insertion of anisotropic defects while thermal expansion is defined by anisotropy of elastic stiffness and Grüneisen parameters. Structural deformations induced by temperature (‘phonon pressure’) and radiation damage (‘defect pressure’) are not necessarily correlated with each other. For two out of three materials studied by McMonagle *et al.* (2024 ▸), deformations induced by phonon and defect pressure were directionally different from each other. To determine the exact nature of the defects one would want to look at the local and not an average structure. The authors supposed that different bonds and intermolecular contacts are affected by anharmonic phonons and radiation-induced defects. Therefore, thermal and radiative expansion have different underlying structural mechanisms.

These findings should be taken into account when studying thermal expansion with synchrotron light, since the true picture may be ‘masked’ by the radiation-induced strain. But the importance of this work is more significant than that. Many physical properties of small-molecule crystals, in particular their ferroelectric and piezoelectric phase transitions, are related to structural strain on temperature or pressure variation. Synchrotron radiation can be also used to manipulate structural strain and in this way it can be used as a tool to affect also the temperature- and pressure-induced phase transitions: to prevent them or to shift the transition point. Examples of the effect of radiation on phase transitions in ferroelectrics have been already documented in the literature (Chernyshov *et al.*, 2022 ▸; Grzechnik *et al.*, 2023 ▸). The paper by McMonagle *et al.* (2024 ▸) shows clearly how more light on the problem may be shed by a quantitative comparison of the anisotropic thermal and radiation expansions. Complementary multi-temperature studies of materials by synchrotron light and at laboratory sources may also be very important in order to reveal radiation strain effects reliably.

## Figures and Tables

**Figure 1:**
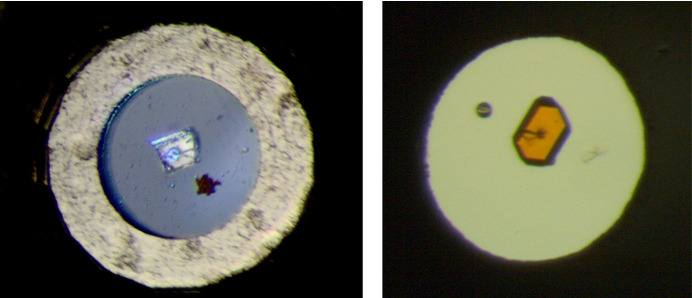
Samples of small-molecule crystals beam-drilled by synchrotron radiation during diffraction experiments. Courtesy of Professor Boris Zakharov.

**Figure 2:**
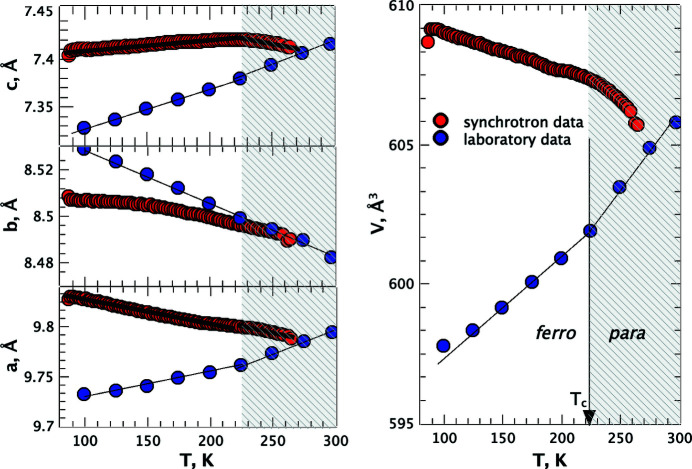
Unit-cell parameters and volume of glycinium phosphite versus temperature from synchrotron and laboratory data. The error bars, if they appear missing, are smaller than the symbols. The shaded zone indicates the temperature domain for the paraelectric phase. The temperature in the experiments changed from the higher to lower values. Reproduced from Bogdanov *et al.* (2021 ▸).

**Figure 3:**
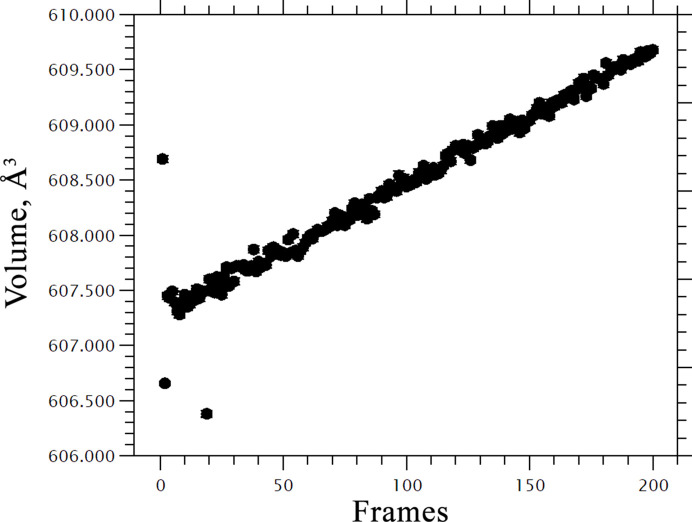
Changes in the unit-cell volume of glycinium phosphite at constant temperature over time resulting from radiation damage. Data collected by Professor Dmitry Chernyshov at SNBL ESRF (Bogdanov *et al.*, 2022 ▸).

**Figure 4:**
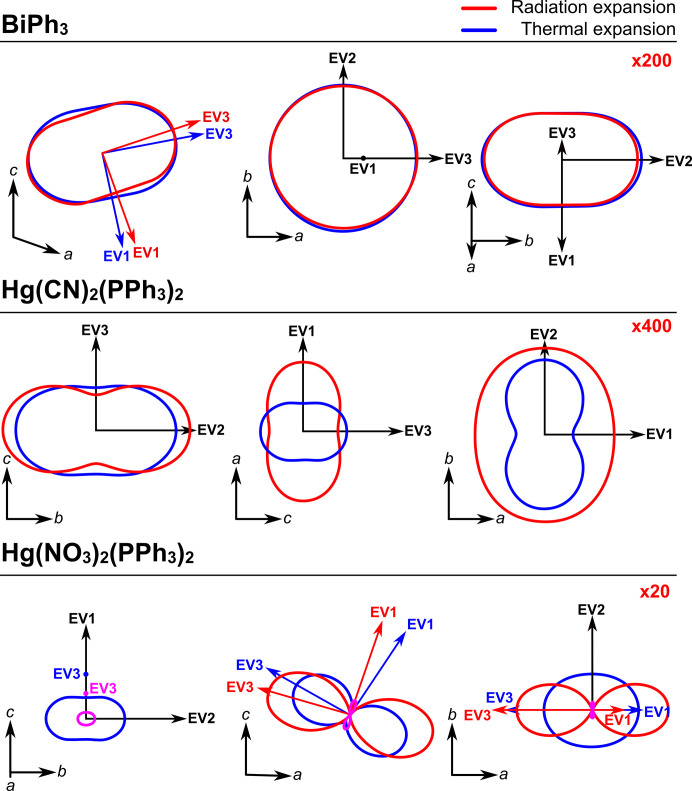
Expansion indicatrices of three compounds. Each panel shows three sections of the indicatrix, viewed along each of the Cartesian coordinates, with the corresponding unit-cell vectors and expansion eigenvectors (EV) labelled. Blue sections show thermal expansion and red the expansion due to radiation. The pink lines indicate negative expansion induced by radiation. Where the thermal and radiation expansion share the same eigenvectors, these are shown in black, otherwise they are shown in blue and red, respectively. To make the thermal expansion (in K^−1^) and radiation expansion (in Gy^−1^) a comparable size in the diagram, the radiation expansion has been multiplied by the figure given in red in the top right of each panel. Reproduced from McMonagle *et al.* (2024) ▸.
